# Complete Genome Sequences of Three *Xanthomonas citri* Strains from Texas

**DOI:** 10.1128/genomeA.00609-17

**Published:** 2017-07-13

**Authors:** Alejandra Munoz Bodnar, Gem Santillana, Vessela Mavrodieva, Zhaowei Liu, Mark Nakhla, Dean W. Gabriel

**Affiliations:** a Department of Plant Pathology, University of Florida, Gainesville, Florida, USA; b United States Department of Agriculture, Animal and Plant Health Inspection Service, Beltsville, Maryland, USA

## Abstract

The complete genome sequences of three *Xanthomonas citri* strains isolated from lime trees in Texas were found to belong to the A^w^ group. All carried nearly identical large plasmids with similarity to those of a citrus canker strain from India and to xanthomonads from Africa and Colombia. All three strains harbored unusual *pthA* homologs.

## GENOME ANNOUNCEMENT

*Xanthomonas citri* subsp. *citri* and *Xanthomonas fuscans* subsp. *aurantifolii* cause identical canker disease symptoms on citrus hosts (1). All pathogenic strains of both species inject a common type III effector (TTE), PthA, into host cells (2). Strains of *X. citri* subsp. *citri* are subdivided into pathotypes A, A*, and A^w^ (3). The A^W^ pathotype is characterized by virulence on lime, determined by the TTEs XopF1 and AvrGF1 (4).

Citrus canker disease was eradicated from Texas in the 1940s. Three *Xanthomonas* strains (160042, 160149, and 160197) were recently isolated from lime trees exhibiting citrus canker symptoms in Texas. PacBio sequencing was used to obtain the complete genomes of these strains, which were 5,330,822 bp, 5,341,733 bp, and 5,337,252 bp in size with 419×, 393×, and 144 × coverage, respectively. Assemblies were performed using SMRT Portal version 2.3 (Pacific Biosciences, Menlo Park, CA), and annotations were generated using PROKKA (5).

The three genomes revealed average nucleotide identity (ANI) values (6, 7) of >99% compared to *X. citri* subsp. *citri* strains 306A and A^W^. Mauve analyses (8) revealed that the highest genome similarities were to A^W^. The TTE repertoire and lipopolysaccharide (LPS) genes of the Texan strains were identical to those described in *X. citri* subsp. *citri* pathotype A^W^, including TTEs *avrGF1* and *xopF1* (4).

Strains 160042 and 160197 had 2 plasmids each, and strain 160149 had 3 plasmids. One of the plasmids in all three strains was large (>123,557 bp) and nearly identical in sequence (>99% ANI) in all three strains. BLASTN (9) revealed high levels of extensive identity with contigs from multiple partial genomes of strains identified as both *X. citri* subsp. *citri* and as *Xanthomonas axonopodis* pv. manihotis. *X. axonopodis* pv. manihotis is not a pathogen of citrus but causes bacterial blight of cassava. The *X. citri* subsp. *citri* strain 160042 large plasmid revealed 99% identity with contigs from both *X. citri* subsp. *citri* strain NCPPB 3562, isolated in India from lemon, and *X. axonopodis* pv. manihotis strain UG21, isolated from cassava in Uganda (10), with 85% and 82% query coverage, respectively. Of note, the plasmids contained a region of ca. 39 kb identified by T346Hunter (11) as similar to type 4 conjugational transfer genes, indicating the potential for horizontal transfer. No genes encoding potential effectors were identified on any of these plasmids. Three primer sets found useful for identifying this plasmid were GAGGACGGGAGGTATGTCCT and AACTCGATGTTGGCTCCCAG, CGGCTACCTCGAAGGATTGG and TTGGTGATTCGTCCTGCTCC, and ACGGAGGTCGGTAAGGAAGT and CTCGGCACACCGTAGATGAA.

All previously described strains of either *X. citri* subsp. *citri* or *X. fuscans* subsp. *aurantifolii* that cause citrus canker encode PthA, a TTE protein with 17.5 tandem, nearly identical, 34-amino-acid (aa) direct repeats (12). Unusually, none of the homologs found in the Texan strains carried 17.5 repeats, but instead, each strain carried one 18.5 repeat version and either a 19.5 or a 14.5 tandem repeat version. Based on the transcription activator-like (TAL)-TTE effector code (13), the 18.5 repeat variants were all predicted to bind genomic target(s) nearly identical to those of the other known functional PthA orthologs, suggesting that these variants represent functional copies of PthA. Also unusually, 160042 and 160197 encoded the predicted functional orthologs on their chromosomes.

### Accession number(s).

These genomes have been deposited in GenBank under accession numbers CP020882 to CP020884 for 160042, CP020885 to CP020888 for 160149, and CP020889 to CP020891 for 160197.

## References

[1] Schaad NW, Postnikova E, Lacy G, Sechler A, Agarkova IV, Stromberg PE, Stromberg VK, Vidaver AK 2006 Emended classification of xanthomonad pathogens on citrus. Syst Appl Microbiol 29:690–695. doi:10.1016/j.syapm.2006.08.001. 17183629

[2] Brunings AM, Gabriel DW 2003 *Xanthomonas citri*: breaking the surface. Mol Plant Pathol 4:141–157. doi:10.1046/j.1364-3703.2003.00163.x. 20569374

[3] Gordon JL, Lefeuvre P, Escalon A, Barbe V, Cruveiller S, Gagnevin L, Pruvost O 2015 Comparative genomics of 43 strains of *Xanthomonas citri* pv. citri reveals the evolutionary events giving rise to pathotypes with different host ranges. BMC Genomics 16:1098. doi:10.1186/s12864-015-2310-x. 26699528 PMC4690215

[4] Jalan N, Kumar D, Andrade MO, Yu F, Jones JB, Graham JH, White FF, Setubal JC, Wang N 2013 Comparative genomic and transcriptome analyses of pathotypes of *Xanthomonas citri* subsp. *citri* provide insights into mechanisms of bacterial virulence and host range. BMC Genomics 14:551. doi:10.1186/1471-2164-14-551. 23941402 PMC3751643

[5] Seemann T 2014 Prokka: rapid prokaryotic genome annotation. Bioinformatics 30:2068–2069. doi:10.1093/bioinformatics/btu153. 24642063

[6] Goris J, Konstantinidis KT, Klappenbach JA, Coenye T, Vandamme P, Tiedje JM 2007 DNA–DNA hybridization values and their relationship to whole-genome sequence similarities. Int J Syst Evol Microbiol 57:81–91. doi:10.1099/ijs.0.64483-0. 17220447

[7] Rodriguez-R LM, Konstantinidis KT 2014 Bypassing cultivation to identify bacterial species. Microbe 9:111–118. doi:10.1128/microbe.9.111.1.

[8] Darling AC, Mau B, Blattner FR, Perna NT 2004 Mauve: multiple alignment of conserved genomic sequence with rearrangements. Genome Res 14:1394–1403. doi:10.1101/gr.2289704. 15231754 PMC442156

[9] Zhang Z, Schwartz S, Wagner L, Miller W 2000 A Greedy algorithm for aligning DNA sequences. J Comput Biol 7:203–214. doi:10.1089/10665270050081478. 10890397

[10] Bart R, Cohn M, Kassen A, McCallum EJ, Shybut M, Petriello A, Krasileva K, Dahlbeck D, Medina C, Alicai T, Kumar L, Moreira LM, Rodrigues Neto J, Verdier V, Santana MA, Kositcharoenkul N, Vanderschuren H, Gruissem W, Bernal A, Staskawicz BJ 2012 High-throughput genomic sequencing of cassava bacterial blight strains identifies conserved effectors to target for durable resistance. Proc Natl Acad Sci U S A 109:E1972–E1979. doi:10.1073/pnas.1208003109. PMC3396514 22699502

[11] Martínez-García PM, Ramos C, Rodríguez-Palenzuela P 2015 T346Hunter: a novel web-based tool for the prediction of type III, type IV and type VI secretion systems in bacterial genomes. PLoS One 10:e0119317. doi:10.1371/journal.pone.0119317. 25867189 PMC4395097

[12] Duan YP, Castañeda A, Zhao G, Erdos G, Gabriel DW 1999 Expression of a single, host-specific, bacterial pathogenicity gene in plant cells elicits division, enlargement, and cell death. MPMI 12:556–560. doi:10.1094/MPMI.1999.12.6.556.

[13] Boch J, Scholze H, Schornack S, Landgraf A, Hahn S, Kay S, Lahaye T, Nickstadt A, Bonas U 2009 Breaking the code of DNA binding specificity of TAL-type III effectors. Science 326:1509–1512. doi:10.1126/science.1178811. 19933107

